# Modeling the clonal heterogeneity of stem cells

**DOI:** 10.1186/1742-4682-7-44

**Published:** 2010-11-17

**Authors:** David P Tuck, Willard Miranker

**Affiliations:** 1 Department of Pathology, Pathology Informatics, Yale University School of Medicine, New Haven, Connecticut 06510, USA; 2 Department of Mathematics, Yale University, New Haven, Connecticut 06520, USA

## Abstract

Recent experimental studies suggest that tissue stem cell pools are composed of functionally diverse clones. Metapopulation models in ecology concentrate on collections of populations and their role in stabilizing coexistence and maintaining selected genetic or epigenetic variation. Such models are characterized by expansion and extinction of spatially distributed populations. We develop a mathematical framework derived from the multispecies metapopulation model of Tilman et al (1994) to study the dynamics of heterogeneous stem cell metapopulations. In addition to normal stem cells, the model can be applied to cancer cell populations and their response to treatment. In our model disturbances may lead to expansion or contraction of cells with distinct properties, reflecting proliferation, apoptosis, and clonal competition. We first present closed-form expressions for the basic model which defines clonal dynamics in the presence of exogenous global disturbances. We then extend the model to include disturbances which are periodic and which may affect clones differently. Within the model framework, we propose a method to devise an optimal strategy of treatments to regulate expansion, contraction, or mutual maintenance of cells with specific properties.

## Background

The promise of therapeutic applications of stem cells depends on expansion, purification and differentiation of cells of specific types required for different clinical purposes. Stem cells are defined by the capacity to either self-renew or differentiate into multiple cell lineages. These characteristics make stem cells candidates for cell therapies and tissue engineering. Stem cell-based technologies will require the ability to generate large numbers of cells with specific characteristics. Thus, understanding and manipulating stem cell dynamics has become an increasingly important area of biomedical research. Genomic and technological advances have led to strategies for such manipulations by targeting key molecular pathways with biological and pharmacological interventions [1-3], as well as by niche or microenvironmental manipulations [4].

Recent conceptual and mathematical models of stem cells have been proposed [5-9] that extend the relevance of earlier ones [10] by focusing on the intrinsic properties of cells and effects of the microenvironment, and address new concepts of stem cell plasticity. Sieburg et al have provided evidence for a clonal diversity model of the stem cell compartment in which functionally discrete subsets of stem cells populate the stem cell pool [11]. In this model, heterogeneous properties of these clones that regulate self-renewal, growth, differentiation, and apoptosis informed by epigenetic mechanisms are maintained and passed onto daughter cells. Experimental evidence supports this notion that tissue stem cell pools are composed of such functionally diverse epigenetic clones [11]. Roeder at al, by extending their previous model to include clonal heterogeneity, have demonstrated through agent based model simulations that clonally fixed differences are necessary to explain the experimental data in hematopoietic stem cells from Sieburg [12].

Metapopulation models concentrate on collections of populations characterized by expansion and extinction and the role of these subpopulations in stabilizing coexistence and maintaining genetic or epigenetic variation. The canonical metapopulation model [13] for the abundance of a single species *p*, with colonization rate *c *and extinction rate *m*, is described by the equation *dp*/*dt *= *cp*(1-*p*) - *mp*. Both the single species model [14,15] and multispecies models have been extensively studied [16-19], identifying various conditions under which effects such as stochasticity of the demographics or the disturbance patterns, spatial effects, habitat size, and asynchronicity, may have theoretical and practical implications, for instance in managing disturbed ecological systems.

The important and influential model of habitat destruction by Tilman [20] extended the multiple species models by including the incorporation of fixed disturbance, conceived as loss of habitat. In the present work, we modify the basic ecological framework from Tilman to model individual cells. Previous metapopulation modeling of individual cellular populations have been proposed. For example, Segovia-Juarez et al, have explained granuloma formation in tuberculosis infections by using simple metapopulation models [21].

The hierarchical structure of the Tilman model is based on a collection of a large number of patches. Each patch can be empty, or inhabited by species *i*. The species are in competition for space and ranked according to their competitive ability. When a cell expands to another patch, it can colonize either if that patch is empty or it is inhabited by species *j *having a lower rank. Analytical studies of the Tilman model have demonstrated that under certain conditions, the species will go extinct according to their competitive ranking. For instance, in the limiting model in which all species have equal mortalities, in the presence of fixed niche destruction, extinction will take place first for the strongest competitors.

We explore the outcome of the interactions of these components using mathematical models. Disturbances in the ecological models refers to externally caused deaths, In the cellular context, they could include the possibility of drug treatments or environmental toxicity. These models are also studied by simulation. In our model the role of individual species is based on individual clones with clonally fixed differences. Increasing evidence is accumulating that cell fate decisions are influenced by epigenetic patterns (such as histone methylation and acetylation status) which may distinguish various clones. Specific gene patterns render different cells uniquely susceptible to differentiation-induced H3K4 demethylation or continued self-renewal [11,22,23].

Unlike the Tilman model, our model treats the generalized case in which each distinct clone can have differing growth and death characteristics. Thus, the strict ordering of extinction does not occur. The model assumes competition for space within a niche among cells with differing growth and self-renewal characteristics.

Expansion and contraction of stem cell populations and the possibility for manipulation of these dynamics will be different for molecular perturbations which target intrinsic growth differentiation or apoptotic pathways or non-specific perturbations. The source of such perturbations is outside of the stem cells themselves, whether from the local microenvironment or from distal locations within the organism such as inflammation, hormonal, cytokine or cell type specific signals (anemia, thrombocytopenia). A related area is the study of subpopulations of cells within tumors that drive tumor growth and recurrence, termed cancer stem cells [24], and which may be resistant to many current cancer treatments [25]. This has led to the hypothesis that effective treatment for such cancers may require specific targeting of the stem cell population.

In this paper, we develop a mathematical framework derived from metapopulation models that can be used to study the principles underlying the expansion and contraction of heterogeneous clones in response to physiological or pathological exogenous signals. In Section 2, we present closed-form expressions for the basic model. We are able to provide closed form analysis of the model near equilibrium states. Combined with numerical simulations, this can provide novel insights and understanding into the dynamics of the phenomena that can be tested experimentally. In Section 3, we explore the effects of both intrinsic cellular characteristics and patterns of exogenous disturbances. In Section 4, we extend the model to include disturbances which may differ quantitatively for different clones. We also extend the analysis from fixed to periodic disturbances. In Section 5, we propose a method to devise an optimal strategy of applying deliberate disturbances to regulate expansion, contraction, or mutual maintenance of specific clones. Finally, in Section 6, we discuss the model and its potential applications.

## A cellular metapopulation model

**Figure 1:**
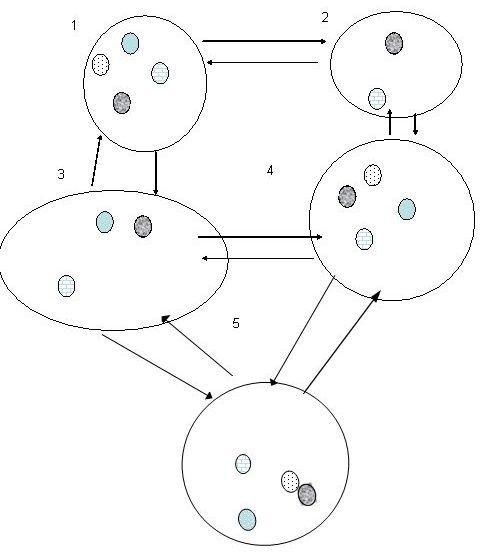
**Metapopulation Concept: Collections of local populations of different clones interact in a niche-matrix view of a microenvironment via dispersal of individuals among niches (large ovals)**. The niches are numbered from 1-5, starting in the upper left. Each niche can be empty, or inhabited by on or more clones i, represented by small shaded ovals. The clones are numbered 1-4 with #1 annotated with cross hatches, #2 with diagonal bricking, #3 with diagonal stripes and #4 with speckles. Arrows depict the movement of clones by migration, extinction and recolonization, as the case may be, within the microenvironment. Despite local extinctions the metapopulation may persist due to recolonization. Suitable niches can be occupied or unoccupied. Metapopulation models are based on niche occupancy over time. Distinct clones with fixed growth characteristics are in competition. Exogenous disturbances (D in Equation 2.1) which deplete specific clones may influence proportions of the surviving clones.

To start, we explore a model of the dynamics of a heterogeneous collection of stem cell clones. Extrapolating from multi-species competition models as well as metapopulation models, our model assumes that clones interact within a localized niche in a microenvironment, and that niches may be linked by cell movements. As in many ecological models, niche occupancy itself, rather than individual cells, is the focus. Figure 1 depicts the cellular metapopulation process in which niches are represented by large ovals, each potentially populated by different clones. Arrows depict the movement of clones by migration, extinction, differentiation, and recolonization, within the microenvironment

Let *R*(*ij*), $i=1,...,\bar{i},\;j=1,...,\bar{j}$ be the occurrency matrix of cell type *j *in niche *i*. For example in Figure 1, number the niches from 1-5, starting in the upper left-most niche (so that $\bar{i}=5$ in this case). The species are numbered 1-4 with #1 annotated with cross hatches, #2 with diagonal bricking, #3 with diagonal stripes and #4 with speckles (so that $\bar{j}=4$ in this case). Then the corresponding occurrency matrix is

$$R(ij)=\left[\begin{array}{cccc} 1 & 1 & 1 & 1\\ 1 & 0 & 0 & 1\\ 1 & 0 & 1 & 1\\ 1 & 1 & 1 & 1\\ 1 & 1 & 1 & 1 \end{array}\right]$$

Next, let

$$p_{j}=\sum_{i}R\left(ij\right)$$

be the number niches containing species $j=1,...,\bar{j}$.

We now present a continuous version of this model in Equation (2.1). For the case of a non-specific perturbation, the dynamics are described by the following differential equations:

(2.1) $$\frac{dp_{i}}{dt}=c_{i}p_{i}\left(1-D-\sum_{j=1}^{i}p_{j}\right)-m_{i}p_{i}-\sum_{j=1}^{i-1}c_{ij}p_{i}p_{j},\;\;i\geq 1$$

Here the *p_i _*denote the number of niches occupied by the *i*-th clone. The *c_i _*denote expansion (or growth) rates, and the *m_i _*extinction (or death) rates. The *c_ij _*represent interactions between pairs of clones. Non-specific niche perturbations, *D*, represent exogenous disturbances which may include pharmacologic, physiologic, or pathologic causes. We extend this, in Section 4, to include clone-specific disturbances, *d_i_*, represent disturbances which have different effects on the various clones.

The behavior of the model is complex; see for example Tilman [20] and Nee [26] for analyses of specific aspects of similar ecological models. We consider a number of simplifications in order to focus on the role of disturbances as deliberate manipulations that alter the expansion and contraction of clones with different fixed characteristics. We consider that each niche is fully connected to all other niches, so that spatial effects are not directly modeled. Similar to the ecological models, we make the hypothesis that clonal lineages have a ranked order in which the abundance of clone *i *within a niche is not affected by clone *j*, but clone *j *is affected by clone *i *(where *i *<*j*). Cells may be removed by either death or by differentiation.

### Nested Switches

This model has been thoroughly analyzed for species abundance in the ecological context of habitat destruction. In ecosystems, the value of D is constantly increased. Analytical studies have revealed conditions which define the order of extinction according to competitive ranking. and the richness or diversity of persisting species and the order of extinction. Such analyses have usually focused on communities with equal mortalities for all species (*m_i _*= *m*) or equal colonization abilities (*c_i _*= *c*). A number of studies have characterized richness or diversity of persisting species and the order of extinction [27-29]. Recent studies have focused on changes in abundance ranking [18]. More recently, Chen et al [30] have assessed the effects of habitat destruction using this model in the presence of the Allee effect. The equilibrium abundances have been studied under a variety of conditions to demonstrate that it is possible, for instance, for species which are not the best competitor to go extinct first if its colonization rate satisfies certain conditions.

We build on these previous analyses and analyze the case allowing both different mortalities and colonization rates for different clones. In this analysis, there is no fixed order of extinction, but rather we demonstrate the existence of a mathematical construct (2.6) that expresses the switching ability among potential states of the system based on differences in the disturbance. Thus, the disturbance, which represented habitat destruction in the ecosystem models, is viewed as a treatment, and our aim is to understand how different treatment choices, by modifying D, can lead to different patterns of clonal abundance. These switching possibilities suggest that clones with different characteristics may, in principle, be selected for expansion through directed, purposeful disturbances.

Introducing new variables

(2.2) $$q_{i}=c_{i}p_{i},\;i\geq 1,$$

the dynamics in (2.1) become

(2.3) $$\frac{dq_{i}}{dt}=\alpha_{i}q_{i}-\sum_{j=1}^{i-1}\beta_{ij}q_{i}q_{j}-q_{i}^{2},\;i\geq 1,$$

where

(2.4) $$\alpha_{i}=c_{i}(1-D)-m_{i},\;i\geq 1,$$

and

(2.5) $$\beta_{ij}=\frac{c_{i}+c_{ij}}{c_{j}}$$

In Appendix A, using (2.3),(2.4), (2.5), we derive the following expression that displays the nested switching.

(2.6) qi∞=[αi−∑j=1i−1βjiqj∞]+, i≥1 =[αi−β1i[α1]+−β2i[α2−β12[α1]+]+−⋯  −βi−1,i[αi−1−β1,i−1[α1]+−⋯−βi−1,i−1[αi−2−β12[αi−1]+]+⋯]+]+,︸i i≥1.

This shows that the equilibria $q_{i}^{\infty }$ have 2^*i *^states among which they might switch. Identification of such a set of nested switches allows us to adjust the model parameters to control expansion or contraction of individual clones. In Section 3 we examine these switchings in terms of the original variables.

### Stability

The model has been widely studied in ecology. For instance, analysis of the stability of an earlier version of this model was provided by Nee [26], and detailed analysis of equilibria performed by Tilman [20,31]. Tilman et al [32] expanded the analysis to a number of variants,based on the initial abundance and different mortality rates for better competitors. Morozov et al study the model analytically to assess changes in abundance ranking over time [18]. Other variations have also been studied including Allee effect's influence on species extinction order [30].

Our analysis of the model includes some minor modifications from previous analyses: each clone may have a different mortality and the interaction between pairs of clones is distinct (*c_ij _*matrix). In the Appendix B we show that the steady state solutions *q_i_*, *i *≥ 1 of (2.3) are unconditionally asymptotically stable with the equilibrium values given in (2.6). This stability combined with the pattern of nested switches suggests that within the scope of the model, we can define predictable interventions either untargeted (based on alterations of non-specific exogenous disturbances) or targeted (based on the growth and death properties of specific clones). Moreover, the nature of the nested switches suggests that clones with different patterns of potential for self-renewal or differentiation may in principle be selected for expansion or contraction by intervening to modify specific or non-specific targets.

### Simulation of the dynamics

**Figure 2:**
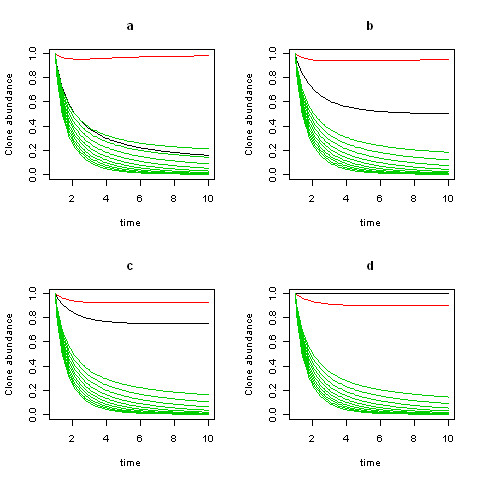
**a-d: Solutions for (2.3) are displayed for three clones (black: clone 1, red: clone 2, green: clone 3)**. A different value of *α*_1 _is used in each panel (a: 0.10, b: 0.50, c: 0.75, d: 1.0). A fixed value *α*_2 _= 1 is used throughout, and a range of values (0.1, 0.2, 0.3, 0.4, 0.5, 0.6, 0.7, 0.8, 0.9, 1.0) is used for *α*_3_. *β*_21 _= 0.1, *β*_31 _= 0.1, and *β*_32 _= 0.2 throughout.

**Figure 3:**
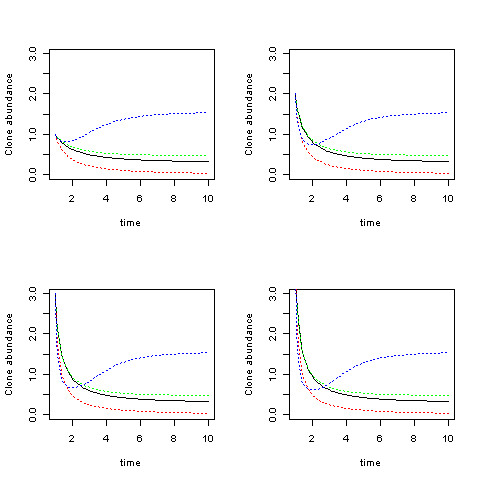
**Solutions of the basic model for four clones (represented by the four different colors) with varying initial values (1,2,3,4) in the different panels**. Fixed values of *α*_1_, *α*_2 _, *α*_3 _, *α*_4_, (0.3,0.5,0,2,0,3) and *β_ij _*= (0.3,0.5,0,2,0,3, 0.1, in lexigraphic order with *j *<*i *< 4) ) are used throughout.

Numerical solutions of (2.3), displayed in Figure 2, affirm both the equilibrium values (2.6) as well as the unconditional stability. Thus, the model predicts the distribution of the clonal populations given functional characteristics of growth and death rates and interaction parameters of a set of clones and a given exogenous disturbance state. Figure 3 shows the different routes to the same limiting equilibrial values and confirms the asymptotic stability in a four clone model.

## Dynamics in terms of the cellular parameters

We now describe the dynamics in terms of the original variables of growth and death rates. In the simplest case of a single clone, the survival of the clone in isolation is determined by the value of *α*_1 _= *c*_1_(1-*D*) - *m*_1 _- see the schematic in (3.1). In the absence of disturbance D, this is simply the canonical single species Levins model, *dp*/*dt *= *cp*(1 - *p*) - *mp*, in which the metapopulation will persist only if m < c. In case a disturbance is present, we see that the clone will survive if the death rate *m *< c(1-D) (shown as the region I of *α*_1 _in (3.1)).(3.1)

From (A.4) we have

(3.2) $$q_{1}^{\infty }={\left[\alpha_{1}\right]}^{+}=\{\begin{array}{cc} \alpha_{1}, & \alpha_{1}\in I\;\\ 0, & \alpha_{1}\in II. \end{array}$$

**Figure 4:**
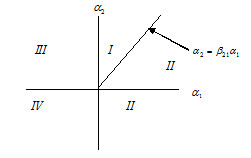
**This schematic shows the plot of *α*_1 _versus *α*_2 _for the two clone model**. For the population pairs ($p_{1}^{\infty },p_{2}^{\infty }$): both clones will survive in domain I, only one ($p_{1}^{\infty }$) survives in domain II, only one ($p_{2}^{\infty }$) survives in domain III, and neither survives in domain IV.

The situation in which there are multiple clones with different growth and death characteristics is a direct extension of this (Figure 4). Note that the straight line segment *α*_2 _= *β*_21_α_1 _in Figure 4 is derived from the switching state [α_2 _- *β*_21_α_1_]+ = 0 (see (2.6)).

In particular, referring to Figure 4, the case of two clonal populations is

(3.3) $$\left(c_{1}p_{1}^{\infty },c_{2}p_{2}^{\infty }\right)=\{\begin{array}{cc} \left(\alpha_{1},\alpha_{2}-\beta_{21}\alpha_{1}\right), & \left(\alpha_{1},\alpha_{2}\right)\in I\\ \left(\alpha_{1},0\right), & \left(\alpha_{1},\alpha_{2}\right)\in II\\ \left(0,\alpha_{2}\right), & \left(\alpha_{1},\alpha_{2}\right)\in III\\ \left(0,0\right), & \left(\alpha_{1},\alpha_{2}\right)\in IV \end{array}$$

We see that for the population pairs ($p_{1}^{\infty },p_{2}^{\infty }$): both clones will survive in domain *I*, only one ($p_{1}^{\infty }$) survives in domain *II*, only one ($p_{2}^{\infty }$) survives in domain *III*, and neither survives in domain *IV*. Thus we have an analytic prescription for the survival or elimination of specific clones. (The equilibrium values in (3.3) in terms of the original variables and the domain descriptions are given in Supplementary Materials). In domain *I*, we have defined conditions for mutual survival of both clones, in domain *II *and *III *we have the selective expansion of the first or second clone, respectively, while in domain *IV*, we obtain extinction of both clones.

### Mutual survival

Some cellular expansion applications might require survival and expansion of some subset consisting of more than one clone. We begin with an example describing in some detail the case in which there are two surviving clones with limiting populations, $p_{1}^{\infty }$ and $p_{2}^{\infty }$. We specify the amount of disturbance that will allow both clones to survive given the growth and death rates and the interaction parameters (the *β*'s). Suppose $q_{1}^{\infty }=\theta q_{2}^{\infty }$, where the constant *θ *> 0. Then from (3.2) and (3.3), we have *α*_1 _= *θ*(α_2 _- *β*_21_α_1_). In terms of the original variables this last relation becomes

(3.4) $$c_{1}\left(1-D\right)-m_{1}=\theta \left(c_{2}\left(1-D\right)-m_{2}-\beta_{21}\left(c_{1}\left(1-D\right)-m_{1}\right)\right).$$

This equation specifies the value of the disturbance D for the survival of both clones, with the relative proportion *θ*, in terms of the cellular parameters. Namely,

(3.5) $$D=1-\frac{m_{1}\left(1+\theta \beta_{21}\right)-\theta m_{2}}{c_{1}\left(1+\theta \beta_{21}\right)-\theta c_{2}}.$$

Note that the only acceptable parameter values are those that deliver positive values for both $p_{1}^{\infty }$ and $p_{2}^{\infty }$. We can extend this analysis to the situation in which there are multiple clones by supposing that

(3.6) $$q_{1}^{\infty }=\theta_{i}q_{i}^{\infty },\;\theta_{i}>0,\;i>1.$$

Using (3.3) and (3.8) this becomes

(3.7) $${\left[\alpha_{1}\right]}^{\;+}=\theta_{i}{\left[\alpha_{i}-\sum_{j=1}^{i-1}\beta_{ij}q_{j}^{\infty }\right]}^{+},\;i>1.$$

In the case in which all the clones survive (that is, each *q_i _*> 0), we may delete the brackets in (3.7), and solve recursively for the *α_i_*. For *i *= 1, the sums in (3.7) are empty, and it yields *θ*_1 _= 0, as expected. For *i *= 2, (3.7) becomes

(3.8) $$\alpha_{1}=\theta_{2}\left(\alpha_{2}-\beta_{21}q_{1}\right),$$

and since from (A.4), *q*_1 _= *α*_1 _(3.8) delivers

(3.9) $$\alpha_{2}=\theta_{2}^{-1}\alpha_{1}+\beta_{21}\alpha_{1}.$$

For three clones in the cellular population we find

(3.10) $$\alpha_{3}=\theta_{3}^{-1}\alpha_{1}+\beta_{31}\alpha_{1}+\beta_{32}\alpha_{2}-\beta_{32}\beta_{21}\alpha_{1}.$$

(Inserting *α*_2 _from (3.9) into (3.10) would allow us to express *α*_3 _in terms of *α*_1_.)

In the general case, the condition for all of an arbitrary number of different clones to survive (in the relative proportion *θ_i _*of *q_i _*to *q*_1_) is derived by extending these arguments. We find

(3.11) $$\alpha_{i}=\theta_{i}^{-1}\alpha_{1}+{\left(-1\right)}^{i}\sum_{n=1}^{i-1}\;\sum_{1\leq k_{1}<\cdots <k_{n-1}<i}\beta_{ik_{i-1}}\prod_{m=1}^{i-2}\beta_{k_{m+1}k_{m}}\alpha_{k_{1}},\;i\geq 1,$$

where *β*'s with undefined subscripts are to be set to unity. Inserting (2.4) into (3.11) we may find the value of the disturbance D that accomplishes the exact degree of mutual survival.

## Oscillations and clone specific disturbance

The model described thus far is limited in that a disturbance to the stem cell microenvironment affects all clonal lineages similarly and does not vary with time. In fact, different disturbances, such as specific cytokine concentration, inflammatory states, proliferative or apoptotic signals from the environment will differentially affect heterotypic cells that are in a particular state at a particular time point. Such perturbations are expected to vary in time with different intensities, durations, and intervals. This scenario could occur in a physiological setting, in which disturbances would occur at different periods over time and in which cell types with different characteristics or in different states of cell cycle, for instance, would respond differentially to these disturbances.

To characterize this situation, we extend the model to include time dependent and population dependent disturbances as follows.

(4.1) $$\frac{dp_{i}}{dt}=c_{i}p_{i}\left(1-D_{i}(t)-\sum_{j=1}^{i}p_{j}\right)-m_{i}p_{i}-\sum_{j=1}^{i-1}c_{ji}p_{i}p_{j},\;i\geq 1$$

For clarity, we take

(4.2) $$D_{i}\left(t\right)=d_{i}-\varepsilon f_{i}(t),\;i\geq 1.$$

Here *d_i _*is a population dependent constant and a harmonic time dependence is taken for the disturbance, namely

(4.3) $$f_{i}(t)=u_{i}\cos\omega_{i}t+v_{i}\sin\omega_{i}t,\;i\geq 1.$$

In this case the *α_i _*of (2.4) become

(4.4) $$\begin{array}{c} \alpha_{i}\left(t\right)=c_{i}\left(1-d_{i}+\varepsilon \;f_{i}(t)\right)-m_{i}\\ \dot{=}a_{i}-c_{i}f_{i}(t)\varepsilon ,\;i\geq 1, \end{array}$$

where *a_i _*= *c_i_*(1 - *d_i_*) - *m_i_*. To avoid confusion, we have denoted the fixed part of *α_i_*(*t*) (namely, the *α_i _*of (2.4)) by the symbol *α_i_*. We seek solutions for the clonal populations in the form of power series expansions in ε. In particular, take

(4.5) $$q_{i}(t)=\sum_{k=0}^{\infty }q_{ik}(t)\varepsilon^{k},\;i\geq 1.$$

In Appendix C, we obtain the following expression for the long time solution of $q_{i}^{\infty }(t)=c_{i}p_{i}(t)$

. The quantities *X_i _*and *Y_i _*are specified in Appendix C.

(4.6) $$q_{i}^{\infty }(t)=q_{i0}^{\infty }\left[1+\varepsilon \frac{c_{i}}{\omega_{i}{}^{2}+b_{i}^{2}}\left(X_{i}\cos\omega_{i}t+Y_{i}\sin\omega_{i}t\right)+O(\varepsilon^{2})\right],\;i\geq 1.$$

Switching effects in (4.6) are expressed within both the $q_{i0}^{\infty }$ and the *b_i_*. The harmonic oscillatory effects are displayed within the parentheses in (4.6)). Then switching and oscillatory effects characterizing $q_{i}^{\infty }(t),\;\;i\geq 1$ are somewhat separate to at least *O*(ε^2^).

**Figure 5:**
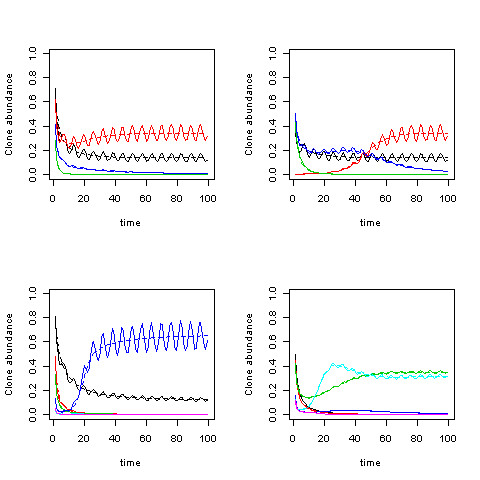
**Four examples of numerical simulations of the population dynamics are displayed**. Results for four clones (different colors) are displayed in the top two panels, with the same parameters except for different initial values. Results for two different simulations are displayed in the bottom two panels. The dynamics can be quite complex. The steady state values in each case correspond to the mean values of the oscillations. Dynamics with oscillating disturbances are displayed in solid lines, while broken lines are used for fixed disturbances. *β_ij _*was randomly selected for each run in the range [0, 1].

Numerical simulations of the oscillatory dynamics for four species (Figure 5) reveal that while the dynamics can be quite complex, the equilibria are stable.

## Single and multiple component perturbation

Having examined the effect of different patterns of disturbances on clonal proportions, we now show how the model may be used to implement clonal expansion or clonal elimination as in cancer applications. We explore the clonal makeup of a population of functionally diverse stem cell clones under different regimens of disturbance. Here, disturbances may be deliberately applied treatments intended to lead to a specific set of clonal proportions. The objective is to find the permissible values of the disturbance parameter *D *so that any specified combination of species survives (including none). In *n*-dimensions there are 2^*n *^such combinations, some of which impose constraints on the model parameters. In Section 5.1, we address the case of a single disturbance that affects all clones in a similar manner. In Section 5.2, we address the use of multiple disturbances that have differential effects on the various clones. For clarity, we shall only display the results of the one and the two species cases (one and two dimensions). In Section 5.3, we illustrate the steps necessary to extend the analysis to three or more species.

### Single intervention, single species protocols

There are 2 possibilities in the case of a single species: (1) survival (2) annihilation.

**(1) **$q_{1}^{\infty }>0$

In this case we have from (A.4) that

(5.1) $$0<q_{1}^{\infty }={\left[\alpha_{1}\right]}^{+}={\left[c_{1}(1-D)-m_{1}\right]}^{+}.$$

Equivalently

(5.2) $$0<U_{1}{\left[-D+A_{1}\right]}^{+},$$

where

(5.3) $$U_{1}=c_{1}\;\text{and}\;A_{1}=1-\frac{m_{1}}{c_{1}}.$$

Since *U*_1 _= *c*_1 _> 0, we can cancel it from (5.2). Then the single species in question survives if the following constraint is imposed on the model's parameters.

(5.4) $$A_{1}>0.$$

Combining this with the requirement *D *≥ 0 gives the following condition on *D*.

(5.5) $$0\leq D<A_{1}.$$

**(2) **$q_{1}^{\infty }\leq 0$

In this case we reverse the inequality in (5.2) to find that

(5.6) $$A_{1}\leq D.$$

Since *D *≥ 0, we write this condition as

(5.7) $${\left[A_{1}\right]}^{+}\leq D.$$

### Single intervention protocols, two species

There are 4 possibilities for two species: (1) both survive, (2) neither survives, (3) only the first is annihilated, and (4) only the second is annihilated.

**(1) **$q_{1}^{\infty }>0$ and $q_{2}^{\infty }>0$

From the 1-dimensional case we have the constraint (5.4) and the condition (5.5) to insure that $q_{1}^{\infty }>0$ To require that $q_{2}^{\infty }>0$, we use (A.4) and append the following inequality to (5.1).

(5.8) $$0<q_{2}^{\infty }={\left[\alpha_{2}-\beta_{21}{\left[\alpha_{1}\right]}^{+}\right]}^{+}.$$

Since (5.4)-(5.5) hold, we may drop the inner plus superscript in (5.8) and rewrite it as

(5.9) $$0<{\left[U_{2}\left[-D+A_{2}\right]\right]}^{+},$$

where

(5.10) $$U_{2}=c_{2}-\beta_{21}c_{1}$$

and

(5.11) $$A_{2}=1-\frac{m_{2}-\beta_{21}m_{1}}{U_{2}}.$$

There are three cases here: (i) *U*_2 _> 0, (ii) *U*_2 _< 0 and (iii) *U*_2 _= 0.

(i) *U*_2 _> 0: In this case, (5.9) becomes [-*D *+ *A*_2_]^+ ^> 0. Combining this with the requirement (5.5) for one-dimension, gives the following range of permissible values for *D*.

(5.12) $$0\leq D<\min\left(A_{1},A_{2}\right).$$

In addition the constraint (5.4) is altered to read

(5.13) $$0<\min\left(A_{1},A_{2}\right).$$

(ii) *U*_2 _< 0: In this case, (5.9) becomes -*D *+ *A*_2 _< 0. Combining this with the requirement (5.5) for one-dimension, gives the following range of permissible values of *D*.

(5.14) $${\left[A_{2}\right]}^{+}<D<A_{1}.$$

This imposes the following constraint on the model's parameters.

(5.15) $${\left[A_{2}\right]}^{+}<A_{1}.$$

(iii) *U*_2 _= 0: In this case, we see from (5.8) and (5.9) that $q_{2}^{\infty }=0$ directly.

**(2) **$q_{1}^{\infty }\leq 0$ and $q_{2}^{\infty }\leq 0$

To annihilate $q_{1}^{\infty }$, we have the condition (5.7) from the one-dimensional case. This requires discarding the inner bracket in (5.8). Then to annihilate $q_{2}^{\infty }$, we have the requirement [α_2_]^+ ^≤ 0, or from (2.4)

(5.16) $$c_{2}\left(-D+1-\frac{m_{2}}{c_{2}}\right)\leq 0.$$

This gives the condition

(5.17) $${\left[{\bar{A}}_{2}\right]}^{+}\leq D,\;\text{where}\;{\bar{A}}_{2}=1-\frac{m_{2}}{c_{2}}.$$

Combining this with (5.7) gives following constraint on the model parameters.

(5.18) $$\max\left({\left[A_{1}\right]}^{+},{\left[{\bar{A}}_{2}\right]}^{+}\right)\leq D.$$

If *c*_2 _= 0, (5.16) shows that (5.17) is not required, and so, (5.18) reduces to (5.7).

**(3) **$q_{1}^{\infty }\leq 0$ and $q_{2}^{\infty }>0$

The condition (5.7) annihilates $q_{1}^{\infty }$. This requires discarding the inner bracket in (5.8), from which we then see that for $q_{2}^{\infty }$ to survive, we reverse the inequality in (5.16). This gives

(5.19) $$0<c_{2}\left(-D+1-\frac{m_{2}}{c_{2}}\right).$$

This requires that *c*_2 _≠ 0 and leads to the following constraint on the model's parameters.

(5.20) $${\bar{A}}_{2}>0.$$

Combining the last two relations gives the condition

(5.21) $$0\leq D<{\left[{\bar{A}}_{2}\right]}^{+}.$$

Combining this with (5.7) gives the condition

(5.22) $${\left[A_{1}\right]}^{+}\leq D<{\left[{\bar{A}}_{2}\right]}^{+}.$$

This imposes the following constraint on the model's parameters.

(5.23) $${\left[A_{1}\right]}^{+}<{\left[{\bar{A}}_{2}\right]}^{+}.$$

**(4) **$q_{1}^{\infty }>0$ and $q_{2}^{\infty }\leq 0$

(5.4) and (5.5) assure that $q_{1}^{\infty }$ survives. In this case we may drop the superscript plus on the inner bracket in (5.8). Then the annihilation of $q_{1}^{\infty }$ requires that the inequality in (5.9) be reversed, giving

(5.24) $${\left[U_{2}\left[-D+A_{2}\right]\right]}^{+}\leq 0.$$

This reverses the two 2-dimensional cases (1)(i) and (ii), which combined with (5.5) gives

(i) *U*_2 _> 0:

(5.25) $${\left[A_{2}\right]}^{+}<D<A_{1},$$

with the following constraint on the model's parameters.

(5.26) $${\left[A_{2}\right]}^{+}<A_{1}.$$

(ii) *U*_2 _< 0:

(5.27) $$A_{2}<D<A_{1},$$

with the following constraint on the model's parameters.

(5.28) $$A_{2}<A_{1}.$$

Finally, (iii) *U*_2 _= 0: (5.24) shows that $q_{2}^{\infty }$ cannot survive.

### Multiple treatment, single species protocols

In the treatment of cancer as well as in expansion of stem cells, desirable results require combinations of treatments. However, these combinations are generally unknown. We propose that this model can be used to derive optimal combinations of treatment, which take the role of disturbances. Although, methods to determine these combinations are various, we demonstrate the feasibility of the approach using a linear programming method [33].

For multiple treatments we replace the *D *in the definition of *α_i _*in (2.4) by *D_i_*. Then with the vector *d *= (*d*_1_, ..., *d_g_*), where *g *is the number of treatments, we write *D_i_*, (the inner product, scalar quantity), as

(5.29) $$D_{i}=\left(F_{i},d\right)=\sum_{j=1}f_{ij}d_{j.}$$

Here the *d_j_*, *j *= 1,2 ..., *g *are quantities of the different treatments used and the vector *F_i _*= (*F_ij_*), *j *= 1,2..., *g*, where *f_ij _*is the efficacy of treatment *j *on species *i*. Each treatment quantity *d_j _*has a collective cost that we call *k_j_*. The objective is to minimize the total treatment cost. Many expressions for the cost may be composed. For clarity, and illustrative purpose, we use the form (*K,d*) where *K *= (*k*_1_, ..., *k_g_*). This requires solving

(5.30) $$\underset{d}{\min}\left(K,d\right)=\underset{d}{\min}\sum_{j=1}^{g}k_{j}d_{j},\;d_{j}\geq 0,$$

subject to certain linear constraints that we shall now assemble. (Such a problem is called a linear program, i.e., minimizing a linear form by varying exogenous parameters (such as *d_j _*in 5.30), subject to linear constraints on those parameters (such as in 5.31, below)) ^33^).

More general, cost expressions would lead to a higher dimensional optimization or a non-linear optimization, any of which could, in principle, be dealt with computationally. With a single species we carry over the constraint (5.4) and the condition (5.7) to the following cases of (1) survival or (2) annihilation.

**(1) **$q_{1}^{\infty }>0$

From (5.5) with *D *replaced by *D*_1 _and from (5.29) we have the condition on the inner product (scalar quantity)

(5.31) $$0\leq \left(F_{1},d\right)<A_{1}.$$

From (5.4), we carry over the following constraint on the model parameters.

(5.32) $$0<A_{1}.$$

**(2) **$q_{1}^{\infty }\leq 0$

Here from (5.7), we have the condition

(5.33) $${\left[A_{1}\right]}^{+}\leq \left(F_{1},d\right).$$

### Multiple treatment, two species protocols

The model allows the extension to multiple species in a straightforward manner. There are four possible states which may be attained by combining treatments for two species.

**(1) **$q_{1}^{\infty }>0$ and $q_{2}^{\infty }>0$

Condition (5.31) and constraint (5.32) insure $q_{1}^{\infty }>0$. To deal with $q_{2}^{\infty }>0$, write (5.8) as

(5.34) $$0<{\left[c_{2}\left(-D_{2}+\frac{1-m_{2}}{c_{2}}\right)-\beta_{21}c_{1}{\left[-D_{1}+\frac{1-m_{1}}{c_{1}}\right]}^{+}\right]}^{+}.$$

Since we have arranged that $q_{1}^{\infty }>0$, drop the inner plus superscript and write (5.34) as

(5.35) $$\left(c_{2}F_{2}-\beta_{21}c_{1}F_{1},d\right)\leq 1-m_{2}-\beta_{21}\left(1-m_{1}\right).$$

**Figure 6:**
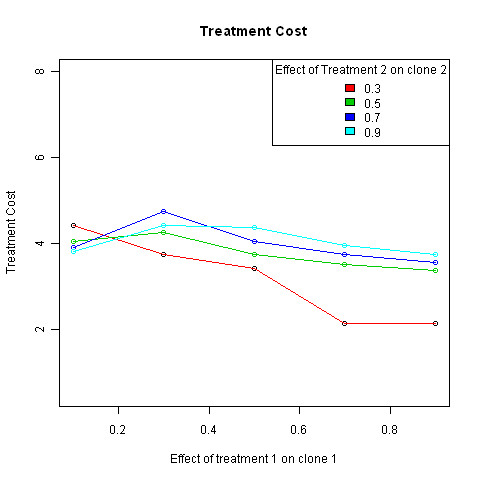
**Solutions to the linear program defined in Section 5.3 identify minimal treatment costs for achieving the desired state of expansion**. We plot the total treatment cost ((K,d) which has been minimized by a linear program in a multi-treatment, two clone model, against a sampling of values of *f*_11 _(the efficacy of the first treatment for the first clone, on the x axis. The results for various values of *f*_22_, the efficacy of the second treatment for the second clone are plotted in different colors. The value of *f*_12 _is set to 0.5. The other parameters have been set to c1 = 0.5, c1 = 0.5, c2 = 0.3, m1 = 0.1, m2 = 0.3, and *β*_21 _= 0.3. The cost per treatment ki is identical for the two treatments. An alternative use of the model would be to determine unknown parameters in an experimental setting where known doses of experimental treatments are applied and outcomes measured in terms of cell proportions.

See Figure 6 for an illustration of the two dimensional case for mutual survival.

**(2) **$q_{1}^{\infty }\leq 0$ and $q_{2}^{\infty }\leq 0$

Condition (5.33) insures that $q_{1}^{\infty }\leq 0$. Then use (5.34) with the inner bracket eliminated and the inequality reversed to insure that $q_{2}^{\infty }\leq 0$. This yields the condition

(5.36) $${\left[{\bar{A}}_{2}\right]}^{+}\leq D_{2}=\left(F_{2},d\right)$$

unless *c*_2 _= 0. In this latter case, we may drop this constraint, since $q_{2}^{\infty }=0$ directly.

**(3) **$q_{1}^{\infty }>0$ and $q_{2}^{\infty }\leq 0$

The condition (5.31) and the constraint (5.32) insure that $q_{1}^{\infty }>0$. Then we reverse the inequality in (5.34) to insure that $q_{2}^{\infty }\leq 0$. This leads to the reversal of the inequality in (5.35). Namely,

(5.37) $$\left(c_{2}f_{2}-\beta_{21}c_{1}f_{1},d\right)>1-m_{2}-\beta_{21}\left(1-m_{1}\right).$$

**(4) **$q_{1}^{\infty }\leq 0$ and $q_{2}^{\infty }>0$

The condition (5.33) insures that $q_{1}^{\infty }\leq 0$. Then we may use (5.34) with the entire inner bracket eliminated to insure that $q_{2}^{\infty }>0$. This leads to the condition

(5.38) $$\left(F_{2},d\right)=D_{2}<{\left[{\bar{A}}_{2}\right]}^{+},$$

unless *c*_2 _= 0, in which case, we may drop this constraint, since $q_{2}^{\infty }=0$ directly.

In Figure 5, solutions to an example set of linear programs are plotted to identify minimal treatment costs for achieving the desired state of expansion. The total treatment cost ((*K,d*) which has been minimized by solving a linear program for each set of parameters in a multi-treatment, two species model, is plotted against a series of values of *f*_11_, the efficacy of the first treatment for the first species, for various values of *f*_22_, the efficacy of the second treatment for the second species. Although the actual methods applied will depend on which parameters are available and which can be estimated, this results demonstrates how the model may be used to determine how to apply specific disturbances to reach a desired outcome.

### Multiple clones

It is straightforward to extend the calculations to the case of three or more clones. We illustrate a single sample case with three clones, namely the case in which only the second clone of three survives (i.e., $q_{1}^{\infty }\leq 0$, $q_{2}^{\infty }>0$, and $q_{3}^{\infty }\leq 0$). We use the constraints in (5.33) and (5.38) to satisfy the first two of these inequalities. To address the third, we use (2.6) to write

(5.40) $$q_{3}^{\infty }={\left[\alpha_{3}-\beta_{31}{\left[\alpha_{1}\right]}^{+}-\beta_{32}{\left[\alpha_{2}-\beta_{21}{\left[\alpha_{1}\right]}^{+}\right]}^{+}\right]}^{+}\leq 0.$$

Since we have arranged that $q_{1}^{\infty }={\left[\alpha_{1}\right]}^{+}\leq 0$, it is, in fact equal to zero and so we may drop the terms [α_1_]^+ ^in (5.40). Then using (2.3), we write (5.40) as

(5.41) $${\left[c_{3}\left(1-D_{3}\right)-m_{3}-\beta_{32}{\left[c_{2}\left(1-D_{2}\right)-m_{2}\right]}^{+}\right]}^{+}\leq 0.$$

This implies that

(5.42) $$c_{3}\left(1-D_{3}\right)-m_{3}-\beta_{32}{\left[c_{2}\left(1-D_{2}\right)-m_{2}\right]}^{+}\leq 0.$$

The bracketed term here is $q_{2}^{\infty }$ itself, and the latter being positive allows us to drop the superscript plus in (5.42). Thus (5.42) delivers the constraint

(5.43) $$c_{3}-m_{3}-\beta_{32}\left(c_{2}-m_{2}\right)\leq \left(c_{3}F_{3}-\beta_{32}c_{2}F_{2},d\right).$$

## Discussion

The therapeutic use of stem cells is one of the most promising frontiers in biomedical research, and has led to interest in the expansion of specific cells for specific clinical purposes. In this paper, we develop a mathematical framework derived from metapopulation models that can be used to study the principles underlying the expansion and contraction of heterogeneous clones in response to physiological or pathological exogenous signals. We show how strategies involving targeted interventions may be defined to expand or contract clonal populations with specific attributes.

The primary contribution of the model is the application of an existing metapopulation paradigm to a new domain. The model has been widely studied in ecology, incorporating the effects of exogenous disturbances. The Tilman model has been widely studied in the ecological context of habitat destruction. Most studies focused on species abundance. The original simplified model, in which the disturbance is fixed to represent irreversible habitat destruction, revealed conditions which define the order of extinction according to competitive ranking. Such analyses have usually focused on communities with equal mortalities for all species or equal colonization abilities. A number of studies have characterized richness or diversity of persisting species and the order of extinction. More recently, Chen et al [30] have assessed the effects of habitat destruction using this model in the presence of the Allee effect. The equilibrium abundances have been studied under a variety of conditions to demonstrate that it is possible, for instance, for species which are not the best competitor to go extinct first if its colonization rate satisfies certain conditions.

We build on these previous analyses and analyze the case allowing both different mortalities and colonization rates for different clones. In this analysis, there is no fixed order of extinction, but rather we demonstrate the existence of a mathematical construct that expresses the switching ability among potential states of the system based on differences in the disturbance. Thus, disturbances, which represented habitat destruction in the ecosystem models, are viewed as treatments, and our aim is to understand how different treatment choices, i.e., modification of the disturbance, can lead to different patterns of clonal abundance. These switching possibilities suggest that clones with different characteristics may, in principle, be selected for expansion through directed, purposeful disturbances.

The problem of identifying treatments which will contribute to expansion of specific lineages has not been extensively studied. Cortin et al have taken an elegant statistical approach to identifying optimal doses for expansion of megakaryocytes (MK) using cytokine cocktails, based on the design of optimal multifactorial experiments [34]. Perturbations leading to expansion of MK precursors were studied through screening cytokines. They identified a specific set of cytokines that maximized MK expansion and maturation. The group of cytokines included thrombopoietin, stem cell factor, interleukin-6, and interleukin-9 as positive regulators and erythropoietin and interleukin-8 as inhibitors of MK maturation. Flt-3 ligand also contributed to the expansion of MK progenitors. The hypothesis that fixed characteristics of heterogeneous clones could be manipulated for expansion could be tested with such a set of cytokines in the setting of relatively purified hematopoietic progenitors or in a cell line, such as the mouse EML which is a multipotent, stem-like cell line, already demonstrated to contain different cell types [35]. Existing approaches might include isolating these subpopulations and expanding them directly. However, such approaches may not be feasible in all situations, such as the requirement for in vivo manipulation as might be required for treatment of cancer stem cells, or in cases where the phenotypic characteristics of different clones might not be sufficiently understood or available to allow isolation.

Another potential use for the model is cancer stem cells. Studies have identified subpopulations of cells within tumors that drive tumor growth and recurrence [24]. Their resistance to many current cancer treatments, has made targeting the contraction of this population an area of major interest in cancer research. A recent paper from Gupta et al is interesting for the identification of existing (etoposide) and newly identified compounds (especially salinomycin in their breast cancer model) which preferentially target stem cells [36]. They also provide evidence that other compounds commonly used in cancer therapy (such as paclitaxel) may enriching for stem cells by targeting other classes of cells. A model in which a multispecies population of such cells existed could be studied in cell lines by treating with different combinations of compounds. Periodic perturbations (intermittent dosages) are common in cancer, both for theoretical reasons of efficacy and for managing toxicity and would likely be components of such interventions in practice.

The incorporation of perturbations as an aspect of the model provides a mechanism for the identification of interventions which can be utilized to expand or contract specific clones with desirable or undesirable growth characteristics. In order to demonstrate the feasibility of the approach, a linear programming approach is outlined as a protocol by means of which optimal doses of multiple interventions are calculated. In practice, values of the necessary parameters are often not known; the model also provides the rationale for an iterative experimental framework in which known doses are applied and the measurement of population sizes and proportions is then utilized to estimate unknown parameters. These estimates can be used as hypotheses to be tested by experimental studies. Growth and death parameters are generally identifiable from existing data. However the interaction among clones is probably more difficult to glean from existing datasets. Therefore an initial application of the model is to determine the interaction values for a set of clones by application of predefined interventions. In addition to the normal stem cells, the model can be applied to the heterogeneity of malignant cells in cancer and responsiveness of such cells to combinations of treatments.

If all the growth parameters of the different clones and their interactions are known, solutions to the linear program can identify optimal doses for each of the treatments that drive the cellular pool into the desired state of expansion. If estimates of the growth parameters are available, a designed experiment with fixed doses of perturbing agents can be applied to determine the minimum costs, for example, at which a specific endpoint can be achieved. An alternative use of the model would be to determine unknown parameters (such as the efficacies of treatments for specific clones, *F_i_*) in an experimental setting where known doses of experimental agents are applied and outcomes measured in terms of cell proportions. These data could then be used to estimate unknown parameters.

The simulations and generalization of the model and its analysis have provided an alternative understanding of clonal heterogeneity. The mathematical framework that includes intrinsic cellular effects, interactions among clones, and exogenous effects within a single model, allows for the possibility that switching, stability, treatment protocols can become tractable features of study.

## Competing interests

The authors declare that they have no competing interests.

## Authors' contributions

DPT and WM conceived of the study, and participated in its design and analysis, and writing of the manuscript. Both authors read and approved the final manuscript.

## Appendix A: Nested Switches

For *i *= 1 the sum in (2.3) is empty, and so the equation for *q*_1 _is decoupled from the system. Writing that equation as $dt=dq_{1}/(\alpha_{1}q_{1}-q_{1}^{2})$ and integrating gives

(A.1) $$t=\int_{q_{1}(0)}^{q_{1}(t)}\frac{dq}{\alpha_{1}q-q^{2}}=-\frac{2}{\alpha_{1}}\tanh^{-1}{\frac{\alpha_{1}-2q}{\alpha_{1}}|}_{q_{1}(0)}^{q_{1}(t)}.$$

Solving (2.6), we find

(A.2) $$q_{1}(t)=\frac{\alpha_{1}}{2}+\frac{\alpha_{1}}{2}\tanh\left[\frac{\alpha_{1}t}{2}-\tanh^{-1}\frac{\alpha_{1}-2q_{1}(0)}{\alpha_{1}}\right].$$

Specifying equilibrium values as

(A.3) $$q_{i}^{\infty }\equiv \underset{t\to \infty }{\lim}\;q_{i}(t),\;i\geq 1,$$

we may observe from (A.2) that

(A.4) $$q_{1}^{\infty }=\frac{\alpha_{1}}{2}\left(1+\mathrm{sgn}\alpha_{1}\right)\equiv \left[\alpha_{1}\right]{\;}^{+}.$$

Here and hereafter we use a standard notation ${\left[x\right]}^{+}=\{\begin{array}{cc} x, & if\;x>0\\ 0, & otherwise \end{array}$.

Now make the equilibrium approximation $q_{j}(t)=q_{j}^{\infty },\;j=1,...,i-1$ in (2.3). This decouples the entire system in (2.3), which becomes

(A.5) $$\frac{dq_{i}}{dt}=\left(\alpha_{i}-Q_{i}\right)q_{i}-q_{i}^{2},\;i\geq 1,$$

where the constants

(A.6) $$Q_{i}=\sum_{j=1}^{i-1}\beta_{ji}q_{j}^{\infty },\;i\geq 1.$$

The decoupling enables (A.5) to be solved for each *q_i_*(*t*) in the closed form (A.2) with *α*_1 _replaced by *α_i _*- *Q_i _*and *q*_1_(0) by *q_i_*(0), *i *≥ 1. Then, in particular, (A.4) gets replaced by

(A.7) $$q_{i}^{\infty }=\frac{\alpha_{i}-Q_{i}}{2}\left[1+\mathrm{sgn}\left(\alpha_{i}-Q_{i}\right)\right]={\left[\alpha_{i}-Q_{i}\right]}^{+},\;i\geq 1.$$

Combining (A.6) and (A.7) recursively gives (identical to equation 2.6):

(A.8) qi∞=[αi−∑j=1i−1βjiqj∞]+, i≥1 =[αi−β1i[α1]+−β2i[α2−β12[α1]+]+−⋯  −βi−1,i[αi−1−β1,i−1[α1]+−⋯−βi−1,i−1[αi−2−β12[αi−1]+]+⋯]+]+,︸i i≥1.

## Appendix B: Stability

To show that the limiting values $q_{i}^{\infty }={\left[\alpha_{i}-Q_{i}\right]}^{+}$ are stable, let

(B.1) $$a_{i}=\alpha_{i}-Q_{i},$$

and make the perturbation

(B.2) $$q_{i}={\left[a_{i}\right]}^{+}+z_{i}.$$

A calculation shows that

(B.3) $$\frac{dz_{i}}{dt}=C_{i}+B_{i}z_{i}-z_{i}^{2},$$

where

(B.4) $$B_{i}=\alpha_{i}-Q_{i}+Q_{i}-\sum_{j=1}^{i-1}\beta_{ji}{\left[a_{i}\right]}^{+}-\sum_{j=1}^{i-1}\beta_{ji}z_{j}-2{\left[a_{i}\right]}^{+}.$$

Here the term -*Q_i _*+ *Q_i _*is appended for convenience. We find in turn that

(B.5) $$B_{i}=-\left|a_{i}\right|-\sum_{j=1}^{i-1}\beta_{ji}z_{j},$$

since |*a_i_*| = *α_i _*- *Q_i _*- 2[*a_i_*]^+ ^and $-\sum_{j=1}^{i-1}\beta_{ji}{\left[a_{i}\right]}^{+}+Q_{i}=0$ by definition. Likewise we find that

(B.6) $$C{\text{​}}_{i}={\left[a_{i}\right]}^{+}\left(\alpha_{i}-Q_{i}-{\left[a_{i}\right]}^{+}+Q_{i}-\sum_{j=1}^{i-1}\beta_{ji}{\left[a_{i}\right]}^{+}\right)$$

vanishes. This is because the last two terms in the parenthesis cancel, while the first two equaling *a_i _*cancel the third for *a_i _*≥ 0. For *a_i _*< 0, the leading factor in (B.6), [*a_i_*]^+ ^= 0. We continue by induction.

For *i *= 1, (B.3) becomes,

$$\frac{dz_{1}}{dt}=-\left|a_{1}\right|z_{1}-z_{1}^{2}$$

whose solution is

(B.7) $$t=\{\begin{array}{l} \frac{2}{\left|a_{1}\right|}\tanh^{-1}\frac{2z_{1}+\left|a_{1}\right|}{\left|a_{1}\right|}+const.,\;a_{1}\neq 0\\ \frac{1}{z_{1}}+const.,\;a_{1}=0. \end{array}$$

From this we see that $\underset{t\to \infty }{\lim}z_{1}=0$, giving unconditional global stability for *q*_1_(*t*).

If this stability has been established for *q_j_*(*t*), for all *j *≤ *i*, the equation for *z*_*i*+1_, may be written as

(B.8) $$\frac{dz_{i+1}}{dt}=\left(-\left|a_{i+1}\right|+o\left(1\right)\right)z_{i+1}-z_{{}_{i+1}}^{2}.$$

The solution of which is

(B.9) $$t=\{\begin{array}{l} \frac{2}{\left|a_{i+1}\right|}\tanh^{-1}\frac{2z_{i+1}+\left|a_{i+1}\right|+o\left(1\right)}{\left|a_{i+1}\right|}+const.,\;a_{i+1}\neq 0\\ \frac{1}{z_{i+1}+o\left(1\right)}+const.,\;a_{i+1}=0. \end{array}$$

From this we see that $\underset{t\to \infty }{\lim}z_{i+1}=0$, completing the induction.

## Appendix C: Switching Effects with Oscillations

Insert (4.3)-(4.5) into (4.1), and collect terms in powers of ε. Then setting the coefficient of ε*^k ^*in what results to zero, we find the following differential equations for the coefficients *q_ik _*in the expansion in (4.5).

(C.1) $$\frac{dq_{ik}}{dt}=a_{i}q_{ik}-c_{i}f_{i}(t)q_{i,k-1}-\sum_{j=1}^{i-1}\beta_{ji}\sum_{l=0}^{k}q_{i,k-l}q_{jl}-\sum_{l=0}^{k}q_{i,k-1}q_{il},\;i\geq 1,\;\;k\geq 0.$$

For *k *= 0, (4.6) yields

(C.2) $$\frac{dq_{i0}}{dt}=\left(a_{i}-\sum_{j=1}^{i-1}\beta_{ji}q_{j0}\right)q_{i0}-q_{i0}^{2},\;i\geq 1,$$

which is the same as (2.3) of Section 1 with *a_i _*replacing *α_i_*. Then referring to (B.5)-(B.7), we find for the limiting equilibrium value $q_{i0}^{\infty }$ of *q*_*i*0_(*t*), the analogous nested set of switches as for the $q_{i}^{\infty }$ in (B.7). Namely

(C.3) $$q_{i0}^{\infty }={\left[a_{i}-\sum_{j=1}^{i-1}\beta_{ji}q_{j0}^{\infty }\right]}^{+},\;i\geq 1.$$

Note in particular that (D.3) gives (compare (B.4))

(C.4) $$q_{10}^{\infty }={\left[a_{1}\right]}^{+}.$$

The case treated in Section 2 corresponds to the leading term in the expansion in (4.5), since when ε = 0, (4.4) gives *α_i _*= *a_i_*. Continuing, we see that for *k *= 1, (D.1) becomes

(C.5) $$\frac{dq_{i1}}{dt}=a_{i}q_{i1}-c_{i}q_{10}^{\infty }f_{i}(t)-\sum_{j=1}^{i-1}\beta_{ji}\sum_{l=0}^{1}q_{i,1-1}q_{jl}-\sum_{l=0}^{1}q_{i,1-1}q_{il},\;i\geq 1.$$

For *i *= 1, the leftmost sum in (D.5) is empty. Then replacing *q*_10 _in (D.5) by its asymptotic value $q_{10}^{\infty }$ (as given in (D.4)) yields

(C.6) $$\frac{dq_{11}}{dt}=b_{1}q_{11}-c_{i}q_{10}^{\infty }f_{1}(t),$$

where

(C.7) $$b_{1}=a_{1}-2q_{10}^{\infty }.$$

The solution of (D.6) is

(C.8) $$q_{11}(t)=e^{b_{1}t}q_{11}(0)-c_{1}q_{10}^{\infty }e^{b_{1}t}\int_{0}^{t}e^{-b_{1}\tau }f_{1}(\tau )d\tau .$$

Using (D.4), note that

(C.9) $$b_{1}=a_{1}-2{\left[a_{1}\right]}^{+}=-\left|a_{1}\right|\leq 0.$$

Using (4.3) and performing the integration in (D.8), we find

(C.10) $$q_{11}(t)=e^{b_{1}t}q_{11}(0)-\frac{c_{1}q_{10}^{\infty }\omega_{1}^{2}}{\omega_{1}^{2}+b_{1}^{2}}\left[\frac{f_{1}(t)}{\omega_{1}}+\frac{v_{1}}{\omega_{1}}e^{b_{1}t}\right]-\frac{b_{1}c_{1}q_{10}^{\infty }}{\omega_{1}^{2}+b_{1}^{2}}f_{1}(t).$$

Then taking the limit (large *t*) here, we find (since *b*_1 _< 0) the following asymptotic form for *q*_11_(*t*).

(C.11) $$q_{11}^{\infty }(t)=\frac{c_{1}q_{10}^{\infty }}{\omega_{1}^{2}+b_{1}^{2}}\left[X_{1}\cos\omega_{1}t+Y_{1}\sin\omega_{1}t\right],$$

where

(C.12) $$X_{1}=\omega_{1}v_{1}-b_{1}u_{1},$$

and

(C.13) $$Y_{1}=-\omega_{1}u_{1}-b_{1}v_{1}.$$

In the general case (employing the established asymptotic forms), (D.5) may be written as

(C.14) $$\frac{dq_{i1}}{dt}=b_{i}q_{i1}-c_{i}q_{i0}^{\infty }F_{i}(t),\;\;i\geq 1,$$

where

(C.15) $$b_{i}=a_{i}-\sum_{j=1}^{i-1}\beta_{ji}q_{j0}^{\infty }-2q_{i0}^{\infty }$$

and

(C.16) $$F_{i}(t)=f_{i}(t)+\frac{q_{i0}^{\infty }}{c_{i}}\sum_{j=1}^{i-1}q_{j1}^{\infty }(t).$$

Referring to (D.9), we can show that all of the *b_i _*≤ 0 by inserting (D.3) into (D.15). Namely,

(C.17) $$\begin{array}{c} b_{i}=a_{i}-\sum_{j=1}^{i-1}\beta_{ji}q_{j0}^{\infty }-2{\left[a_{i}-\sum_{j=1}^{i-1}\beta_{ji}q_{j0}^{\infty }\right]}^{+},\;i\geq 1\\ =-\left|a_{i}-\sum_{j=1}^{i-1}\beta_{ji}q_{j0}^{\infty }\right|,\;i\geq 1. \end{array}$$

Referring to (D.4), assume, using induction, that

(C.18) $$q_{j1}^{\infty }=c_{j}q_{j0}^{\infty }\left[X_{j}\cos\omega_{j}t+Y_{j}\sin\omega_{j}t\right]\;,\;j\leq i-1,$$

where the *X_j _*and the *Y_j _*are to be specified. Inserting (D.18) into (D.16), and then inserting the resultant expression for *F_i_*(*t*) into (D.13), the latter becomes

(C.19) $$\frac{dq_{i1}}{dt}=b_{i}q_{i1}-c_{i}q_{i0}^{\infty }\left(U_{i}\cos\omega_{i}t+V_{i}\sin\omega_{i}t\right),$$

where

(C.20) $$U_{i}=u_{i}+\frac{1}{c_{i}}\sum_{j=1}^{i-1}\beta_{ji}X_{j},$$

and

(C.21) $$V_{i}=v_{i}+\frac{1}{c_{i}}\sum_{j=1}^{i-1}\beta_{ji}Y_{j}.$$

Compare (D.19) to (D.6). Then since from (D.17), all *b*_*i *_< 0, analogy to (D.6)-(D.8) allows us to develop the following asymptotic form of *q*_*i*1_(*t*).

(C.22) $$q_{i1}^{\infty }(t)=\frac{c_{i}q_{i0}^{\infty }}{\omega_{i}{}^{2}+b_{i}^{2}}\left[X_{i}\cos\omega_{i}t+Y_{i}\sin\omega_{i}t\right],$$

where

(C.23) $$X_{i}=\omega_{i}V_{i}-b_{i}U_{i},$$

and

(C.24) $$Y_{i}=-\omega_{i}U_{i}-b_{i}V_{i}.$$

This specification of *X_i _*and *Y_i _*completes the induction.

Collecting terms $q_{i0}^{\infty }$ and $q_{i1}^{\infty }(t)$ (the latter from (D.22)), we may write $q_{i}^{\infty }(t)$, the asymptotic form of *q_i_*(*t*) given in (4.5) as (identical to (4.6))

(C.25) $$q_{i}^{\infty }(t)=q_{i0}^{\infty }\left[1+\varepsilon \frac{c_{i}}{\omega_{i}{}^{2}+b_{i}^{2}}\left(X_{i}\cos\omega_{i}t+Y_{i}\sin\omega_{i}t\right)+O(\varepsilon^{2})\right],\;i\geq 1.$$
